# Chilean National Sports Talent Detection System: Influence of Biological Age, Sex, and Geographic Area

**DOI:** 10.3390/jfmk10010006

**Published:** 2024-12-27

**Authors:** Ariel Guevara-Araya, Samuel Curripan-Henríquez, Juan Aguilera-Julio, Ana Antinao-Soto, Oscar F. Araneda

**Affiliations:** 1 Unidad de Proyección Deportiva Nacional, Departamento de Alto Rendimiento, Instituto Nacional de Deportes, Dirección Nacional, Santiago 7500000, Chile; 2 Unidad de Proyección Deportiva Nacional, Departamento de Alto Rendimiento, Instituto Nacional de Deportes, Dirección Regional de O’Higgins, Rancagua 2820000, Chile; samuel.curripan@ind.cl; 3 Unidad de Proyección Deportiva Nacional, Departamento de Alto Rendimiento, Instituto Nacional de Deportes, Dirección Regional de Antofagasta, Antofagasta 1240000, Chile; juan.aguilera@ind.cl; 4 Unidad de Proyección Deportiva Nacional, Departamento de Alto Rendimiento, Instituto Nacional de Deportes, Dirección Regional de La Araucanía, Temuco 4780000, Chile; pamela.antinao.soto@gmail.com; 5 Integrative Laboratory of Biomechanics and Physiology of Effort, (LIBFE), School of Kinesiology, Faculty of Medicine, Universidad de los Andes, Santiago 7620001, Chile

**Keywords:** peak height velocity, adolescents, physical tests, sports talents, biological maturity, bio-banding

## Abstract

The purpose of this study was to assess the methodology for selecting sports talent in the Chilean Promising Athletes Program (CPAP). Methods: The standing broad jump (SBJ), medicine ball chest throw (MBCT), thirty-meter running sprint (RS-30), Agility-T (T-test), and height were analyzed in 3060 schoolchildren from Chile, grouped by sex, age, geographical area (north, central and south) and maturity status at peak height velocity (PHV) in pre-, circa- and post-PHV. Results: Superior differences were found in boys for all age ranges in SBJ, RS-30, T-test, and MBCT (*p* < 0.05). Girls were taller than boys until the age of 12 years (*p* < 0.01) with a reversal of this trend after 13 years old (*p* < 0.001). In geographical grouping, higher scores in SBJ, RS-30, and T-test were found in the central zone for boys and girls (*p* < 0.05). In both sexes, higher scores for the northern zone in MBCT (*p* < 0.01) and height (*p* < 0.05) are observed. Among selected talents, when comparing post-PHV vs. pre-PHV the differences are superior in all tests for post-PHV in girls and for MBCT and height (*p* < 0.05) in boys. In circa-PHV vs. post-PHV in MBCT, T-test, and RS-30 only girls (*p* < 0.05) were superior in post-PHV. For pre-PHV vs. circa-PHV, RS-30, and height (*p* < 0.05), both sexes were superior in circa-PHV. There are differences between talents selected by physical tests and technical aspects (*p* < 0.05). Conclusions: The results show variations in physical and anthropometric tests in schoolchildren by age, sex, geography, and maturity status that should be considered for talent selection.

## 1. Introduction

The detection and identification of sports talent have been increasing over the last decade through government programs and policies that seek to place their respective nations in world sporting events. Currently, several factors that would allow international sporting success are proposed, like the implementation of public policies, financial support, and the development of talent detection systems [1]. This last aspect has generated great interest in sports science, establishing different paradigms regarding early sports specialization, incidence of injuries, and risk factors associated with biological processes and cognitive development of children and young athletes [2,3].

Subjects with sports talent possess qualities and conditions such as anthropometric characteristics, athletic development, decision-making, or leadership to excel in a sports discipline compared to their peers. Moreover, these skills can be perfected in the long term until exceptional sporting performance is achieved [4,5]. Within this referential framework, there are different talent detection models focused on aspects of potential longitudinal follow-up, such as assessments of moderating parameters (anxiety, motivation, family support) and predictors (technical and/or creative skills), among others [4,5,6].

In the process of detecting athletic talent, four main stages are identified: detection, which consists of discovering those who have athletic performance potential and do not practice a specific sport. Identification, in which those who have the potential for a sports discipline are recognized; development, which corresponds to the process of providing the necessary environment to consolidate the athletic potential; and selection, a process in which the most suitable for a sports task in a specific context is captured. These stages are not isolated but rather interconnected, each one contributing progressively to the next in a complex and deeply integrated process [4,5,6].

Within these talent-selective systems, there are differences in the age of selection and specialization, the sport chosen, the number of training hours, and the interaction with other sports [4,6,7]. According to the above, one of the most widely accepted models corresponds to “late sport specialization” to avoid physiological and psychological overload and respect the adaptation periods necessary to achieve optimal performance [7]. According to this model, genetic, environmental, and psychological factors; athlete support programs; and the volume and frequency of training are identified as the determining elements of the process [4,5,6,8]. Furthermore, in this same context, using machine learning, factors have been added and confirmed that allow the identification of sporting talent through the evaluation of performance in stages prior to the all-competitive category, determining the optimal time of specific dedication to the discipline in formative stages [9,10,11]. Although outstanding performance in lower categories of a sport does not ensure success in all-competitive events [6,12], physical and morphological assessment with respect to sex, age, and maturity could be an initial parameter to be applied in the selective process through a multidimensional work [3,6,7,8,13,14], since, during biological development, differences are observed in biomechanical, physiological, and psychosocial aspects that make the process even more difficult [2,6,13,15].

In another aspect, Chile has never previously developed a sports talent detection program, having only researched physical fitness in the school population to identify risk factors and improve the quality of physical education classes. Thus, based on these results, the authorities have designed action plans, compared with other countries in the region, and implemented other government policies [16,17,18]. Subsequently, the physical activity and sports policy was modified [19]. Thus, purpose No. 4 states: “To position Chile in high-level international competition, through the formation and improvement of the practice of conventional and paralympic performance sports.” Among the actions to fulfill this purpose, the national system for detecting sports talent was implemented in 2020, consisting of the application of a battery of physical and anthropometric tests to identify talents in the school system and incorporate them into the sports program of the Chilean National Sports Institute through the CPAP.

In view of the above and considering the evidence in the area of sports talent selection processes, we proposed, using CPAP data during the period 2020 to 2023, the following objectives: to compare the results of the physical and anthropometric tests according to sex, chronological age categories, biological age, and geographic area, in addition to evaluating the potential difference between those incorporated into the program by the values of the physical tests versus those incorporated by the technical criteria of the coach. Finally, we designed the program’s own tables of results, both for use in subsequent processes within the country and for comparative studies with data from other parts of the world.

## 2. Materials and Methods

### 2.1. Design and Participants

This study corresponds to a secondary data analysis of the individual procedures and values of CPAP participants, which the Chilean National Sports Institute requested. As the government funds this program, any member of the country can freely request these data, with the assurance that participants’ sensitive information is protected according to current legislation (see Institutional Review Board Status below for more details).

Description of the sports talent detection system: 3165 Chilean schoolchildren, enrolled in municipal and private subsidized schools, from which outliers, incorrectly tabulated information, and data outside the age interval of analysis were eliminated, constituting a final sample of 3060 schoolchildren (girls *n* = 1243) from Chile and abroad (343), from fifteen regions of Chile (Table 1). The foreign group (133 girls) consisted of Venezuelan (167), Colombian (53), Bolivian (51), Peruvian (29), Haitian (28), and others (15).

For the study of differences by chronological age, the sample was ordered according to the following age ranges (A = 9 to 9.9, B = 10 to 10.9, C = 11 to 11.9, D = 12 to 12.9, E = 13 to 13.9, and F = 14 to 14.9 years old) by boys and girls, respectively. To study the differences between biological ages, 3 groups were established by maturity status at PHV, according to the van der Sluis protocol [20]; pre-PHV (<−0.5 years at PHV), circa-PHV (≥−0.5 years and ≤0.5 years at PHV), and post-PHV (>0.5 years at PHV).

For analysis by geographic area, regions were grouped into 3 categories derived from the classification by climate, natural resources, and topography [21]; Northern Zone = [Arica and Parinacota region to Aconcagua river (33° S), *n* = 1183 (485 girls)], Central Zone = [south bank of the Aconcagua river (33° S) and north bank of the Bio-Bio river (37° S), *n* = 1334 (509 girls)] and Southern Zone = [south bank of the Bio-Bio river (37° S) to the Magallanes region, *n* = 543 (249 girls)].

### 2.2. Methods

- Anthropometry included weight, height, and sitting height, according to ISAK protocol [22]. A digital scale (model SECA 813, Hamburg, Germany, accuracy 0.1 kg) was used to measure body weight. For the recording of height and sitting height, a stadiometer (model SECA 213; Hamburg, Germany, precision 1 mm) and an anthropometric box of 40 × 50 cm were used.
- Maturational state: PHV was estimated in years, according to the protocol proposed by Mirwald RL [23], using height (cm), sitting height (cm), body weight (kg), leg length (cm), and decimal age in years. The equations used were for girls = [−9.376 + 0.0001882 × Leg Length and Sitting Height interaction + 0.0022 × Age and Leg Length interaction + 0.005841 × Age and Sitting Height interaction—0.002658 × Age and Weight interaction + 0.07693 × Weight by Height ratio], while for boys = [−9.236 + 0.0002708 × Leg Length and Sitting Height interaction −0.001663 × Age and Leg Length interaction + 0.007216 × Age and Sitting Height interaction + 0.02292 × Weight by Height].
- Battery of physical assessments. A tape measure (model INGCO HFMT8250, accurate to 0.1 cm) was employed to record field distances. For speed and agility tests, a photocell kit (model Witty–Microgate, Bolzano, Italy, accuracy 0.001 s) was used.
- Thirty-meter running sprint (RS-30): speed was measured in 30 m (m·s^−1^) according to the protocol of Castro-Piñero. J [24]. A start and finish line was drawn in a demarcated area of 35 m long × 1.5 m wide, placing the photocells at the 0 point and at 30 m at an approximate chest height. To begin, the test subject was positioned with both feet behind the start line, asking the test subject to leave a stationary position at maximum speed to the finish line.
- Agility T-test (T-test). It was evaluated according to Negra. Y [25], where four cones were placed in the shape of a T, two cones at a separation of 9.14 m from a starting line, and two located at a perpendicular distance of 4.57 m to the right and left of the second cone. The photocell was placed at the first cone at a separation of 1.5 m and at an approximate distance from the chest of the test subject, and the time was recorded in seconds (s).
- Standing broad jump test (SBJ). The protocol described by Saint-Maurice was used [26]. The subject was asked to execute a horizontal jump seeking the maximum possible distance, and the closest mark to the starting line at the moment of landing was recorded, in which the heel of the supporting foot was used as a reference. Arm swinging was allowed before the execution. The distance is measured in cm.
- Medicine ball chest throw test (MBCT). The protocol proposed by Hackett. D was used [27]. The test is performed sitting on a chair with the back and feet fully supported on the backrest and floor, respectively. The test subject was asked to push a 3 kg medicine ball (Select^®^ profcare medicine ball) from the center of the chest with both hands as far as possible; shoulders and back should remain in contact with the chair. The distance was measured from the point of contact of the ball with the ground to the nearest 5 cm.
- Team of evaluators: A specialized technical team of five members per region was implemented, all students from the third year of the physical education career and a regional technical coordinator, a physical education teacher with experience in field physical evaluations. The applied sports science unit and the sports projection unit, along with protocols and specific technical criteria for each test, trained each regional team member. Each member of the technical team participated in at least three days of evaluations of athletes belonging to CPAP before starting to apply the test battery.

### 2.3. Protocol

After explaining the procedures to the participants, standing and sitting height was determined, as well as body weight. Subsequently, a 20-min warm-up was performed, and then the children were randomly distributed throughout the stations corresponding to SBJ, MBCT, T-test, and RS-30. Each member of the technical team supervised the correct execution of the tests on a non-slip surface. The children were allowed two test attempts, followed by three subsequent attempts with an intertrial interval of approximately 2 min, after which the best result was recorded.

Children with results above the 85th percentile in three or more physical or anthropometric tests were selected for the following stages of pre-selection and selection, who were assessed and classified according to their maturational status (pre-PHV, circa-PHV, and post-PHV), using reference values constructed from 2019 (pilot plan) and the annual updating of databases to date, as the sample size increased. For the final selection process, the initially detected schoolchildren were referred to motor training schools of the National Sports Institute and were followed up weekly by the coordinator of the technical team in charge of each zone. These athletes alternated twice a week with CPAP training, where the trainer in charge of the discipline evaluated their technical skills. After a maximum period of three months, it was finally determined, together with the program’s coaches and technical teams (psychologist, nutritionist, and kinesiologist), the final selection and identification of the talent to enter CPAP. In addition, the expert advice of the respective coaches for their cognitive, volitional, and tactical skills, specific biotypes for a sport, or evaluation in a specific context was considered as an alternative for entering the program [28,29,30]. These athletes entered in the same way as the model described in the previous paragraph and directly into a trial period in CPAP, without being previously referred to the formative sports schools, for after a similar trial period, it was finally decided their incorporation into CPAP.

### 2.4. Statistical Analysis

Descriptive analysis included mean and standard deviation determination and percentile calculations (10th, 15th, 25th, 50th, 75th, 85th, and 90th) by sex, chronological and biological age group for each test. Values three times the interquartile range (IQR) above the third quartile and below the first quartile were identified as extreme and removed from the sample.

To meet the proposed objectives, a factorial ANOVA model was performed for each physical and anthropometric test with independent variables sex and age range (2 × 6) and one-way ANOVA by sex for geographic area and biological age [31]. The normality of the residuals was double-checked through Kernel density plots and symmetry and kurtosis analysis [31,32]. To evaluate the homogeneity of variances, residual plots were used for each model [31] and in moderate sample sizes [32], the Fligner–Killeen test. When significant differences were found in the interaction and main effects, planned contrasts were calculated using the Benjamin–Hochberg correction for the estimated marginal means.

The Mann–Whitney–Wilcoxon test [31] compared groups using technical selection criteria. A 95% confidence interval (CI) and a significance value of *p* < 0.05 were established. The effect size was calculated through the partial eta squared (η^2^): (small ≥ 0.01, medium ≥ 0.06, and large ≥ 0.14) and Cohen’s d: (small > 0.2, moderate ≥ 0.5, large ≥ 0.8, and very large ≥ 1.3) [31,33]. All analyses were performed with R Studio statistical software, version 4.3.2 [34], Jamovi software, version 2.5 [35] for descriptive statistics; and Microsoft Excel for data processing.

To evaluate the reliability of the group of evaluators, a sample of eight members corresponding to the north (*n* = 5) and center of the country (*n* = 3) was selected, who evaluated in duplicate (test-retest) a group of schoolchildren, participants in the talent tests (*n* = 16, 8 per zone), of whom seven were girls. For the variables corresponding to weight, height, and sitting height, the intra-rater and inter-rater (intra-TEM and inter-TEM, respectively) technical error of measurement (TEM) was calculated, according to the method proposed by Ulijaszek S, [36]. For the physical tests, the SBJ and MBCT tests were selected, where reliability was determined through the Bland–Altman method of mean difference analysis.

## 3. Results

### 3.1. Specialized Technical Team

In the anthropometric reliability tests for intra-TEM, the following values were observed: <0.16%, <0.17%, and <0.6% for weight, height, and sitting height, respectively. For the inter-TEM, <0.075 kg, <0.3 cm, and <0.83 cm, respectively, were determined for weight, height, and sitting height. In the Bland–Altman plots for the SBJ and MBCT physical tests, no significant deviations from the confidence limit (±1.96 standard deviations) were observed for each team of evaluators.

### 3.2. Differences by Sex and Age Range

Table 2 indicates a significant interaction between sex and age range in all the physical and anthropometric tests of the factorial ANOVA model. The study of contrasts for height revealed that girls exhibited superior scores in ranges B–C and boys in ranges E–F, with moderate to large effect sizes.

In the lower limb neuromuscular skills tests, T-test, RS-30, and SBJ, superior differences were found in the boys in the six age groups. The effect size was small in A-B-C and changed from moderate to large in D-E-F in all tests.

For the MBCT test, the boys present greater statistical data in the A-C-D-E and F ranges, following the same dynamics of the lower limb tests. However, from the age of 13, the effect size changes from small to very large.

### 3.3. Differences by Geographic Zone

In Table 3, one-way ANOVA models showed significant differences between geographic areas for each physical fitness test and anthropometric data for boys and girls distinctly.

For height in the contrast tests, in the girls, higher values were observed in the northern zone with respect to the southern zone. In the boys, a higher difference was recorded in the northern zone with respect to the central and southern zones. In the center-south contrast, the difference in height is greater in the central zone, and the magnitudes of the effect are small in both sexes.

In the SBJ test, higher values are observed for both sexes in the central zone, in the north-center and center-south contrasts, and in favor of the northern zone in the north–south contrast, with the magnitude of the difference being small to moderate.

The RS-30 test results for boys showed higher differences in the central zone when compared to the north and south, respectively. Additionally, the northern zone exhibited higher values compared to the south. In girls, results indicated that the center zone had an advantage over the north and south. However, the effect size was found to be small in all contrasts for both sexes.

In the T-test, for the boys, shorter times were observed in the central zone for the center-north and center-south contrasts and in the northern zone for the north–south contrast. In the girls, differences are observed in the north-center and center-south contrast with a predominance of the central zone; the magnitude of the difference is small for both sexes.

In the MBCT test in both sexes, there are only superior differences in the northern and central zones, both in relation to the south, with a small to moderate effect size.

### 3.4. Differences by Biological Age

In Table 4, considering those preselected schoolchildren where it was possible to obtain the maturity status (93%), the one-way ANOVA tests by biological age (independent variable) detected differences in all the dependent variables (physical and anthropometric tests) for both sexes, except for the SBJ test, with no changes in the boys.

During the process of the National Talent Detection System 2020–2023, a total of 371 (40.7% girls) schoolchildren (9.2% foreigners) were preselected, considering physical and anthropometric tests above the cut-off percentile, maturity status, and technical criteria of coaches, of whom a total of 67 (27 girls) finally gained admission to the CPAP, corresponding to 2.2% of the sample initially assessed (Table 4 and Table 5).

In the tests of contrasts, for the girls, superior differences were observed in the circa-PHV category when compared with pre-PHV for height, RS-30, and SBJ with effect sizes from moderate to large. When comparing pre-PHV vs. post-PHV categories, differences were observed in all the tests performed, with moderate to very large effect sizes (height and RS-30) in favor of post-PHV. In the circa-PHV vs. post-PHV contrast, there are superior differences in post-PHV for RS-30, T-test, and MBCT.

In the boys, there are superior differences for the circa-PHV group when contrasted with pre-PHV for height, RS-30, T-test, and MBCT with small to moderate effect sizes. In the pre-PHV vs. post-PHV contrast, there are superior differences for post-PHV in height and MBCT with moderate effect sizes. In the circa-PHV vs. post-PHV contrast, no differences were found.

### 3.5. Differences by Selection Criteria

Table 5 shows the comparative analysis of the tested schoolchildren who have been identified as sports talents and have accessed CPAP (67). In both sexes, differences can be seen concerning the selection criteria, such as being superior in the girls identified by physical tests and the results in SBJ, MBCT, RS-30, and T-test compared to those chosen by technical criteria.

In the male sex, there were only differences in favor of selection by physical tests for SBJ and T-test. There are no significant changes between groups for both sexes in terms of height, age, and maturity status.

## 4. Discussion

Considering the geographic dimensions of Chile, there is great variability with respect to climate, natural resources, urbanization, and migrant population [21,37,38], which could have a specific impact, depending on the sector, on the tests performed on the school population at the national level.

### 4.1. Differences by Sex and Geographical Area

With reference to height, there are differences described in developmental biology [39,40,41], where girls have a greater height up to approximately 12 years of age, which would be related to an advanced maturity status and an earlier growth peak [39,41,42]. In the sample studied, boys reached a taller height than girls only from age ranges E and F, a phenomenon mainly associated with an increase in bone mass and hormonal changes typical of biological development [39,42,43], which is finally expressed in a taller height (Table 2).

When comparing the differences between geographic zones, it is intriguing to note that the greatest height was found in the country’s northern region for both sexes. This discovery may be linked to the influx of European migrants who settled in Chile at the beginning of the 19th century or from other Latin American countries who arrived in the last 20 years [37,38]. A similar finding was previously reported by Malina et al., who highlighted the influence of ethnicity on PHV. They found a gradient in decreasing stature from northern Europe with respect to those from southern Europe [15]. This underscores the need to consider the potential influence of this factor in the selection process of athletes, a point that calls for further research as the program presented in this work expands to include more participants from other ethnic groups.

In the neuromuscular skills of the lower limb, the superior differences of the boys, found in all age ranges for the RS-30 test, T-test, and SBJ, would be due to, in the early stages (ranges A-B-C), better motor coordination, type of sports activities, school infrastructure, geography, and socio-cultural factors [44,45,46,47,48] and later, due to an increase in the amount of testosterone and growth hormone during the pubertal stage, which facilitates osteomuscular development [39,41,42], Table 2.

Regarding the geographical area, the greatest differences found in the central area for both sexes could be due to the interaction of genetic and sociodemographic factors [5,44,45,46,47,48], among others. Research in similar age ranges and populations in Europe and America, like the present study, reports dissimilar results according to geography, which would be specific to the type of population [45,46,47].

When comparing children with higher ability (90th percentile), at approximately pubertal ages (E and F range) with respect to the EUROFIT battery [49], in the SBJ test, superior results are observed for both sexes in the European population. However, in comparison with other samples from the same continent [50], the results would be favorable in the present investigation.

These observations demonstrate the great variability that exists between the results of physical tests of different populations, making the athlete’s development specific. Studies in strength and power sports [51] affirm the results, finding variations in the age of maximum performance of the athlete, according to continent and sex, which would respond to different models of sports development.

For the power of the upper limbs, the differences between sexes in the ranges A, C, D, E, and F for the MBCT test show similar trends to other school populations [27], where boys outperform girls. This difference would be due to increases in strength, bone diameters, and muscle mass of the upper body, associated, as in other tests, with changes in biological development [27,39,41,43]. When studying these differences by geographic area, the highest scores in both sexes were recorded in the northern and central areas, which could be related to extracurricular activities or morphological modifications specific to this population [43,44].

The effect of geographical distribution on the results of anthropometric determinations and physical tests, as discussed in this work, can significantly influence the detection of sports talent. From our perspective, the primary factor that explains this phenomenon is the nationality and ethnic origin of the participants. The country has been experiencing a substantial migratory flow from the northern regions of the continent, with 1.6 million migrants out of a total of about 19 million inhabitants by 2023. These migrants often settle temporarily in the north of the country before dispersing throughout the territory. As a result, demographic changes may be influenced by underlying factors not previously considered, such as the new genetic influx and sociocultural and economic factors [15]. The urgency of studying and evaluating these factors in the processes of sports development cannot be overstated [15,45,46,47,48].

### 4.2. Selected Schoolchildren and Maturity Status

Among the schoolchildren who accessed subsequent processes of the talent detection system, it can be seen that, for both sexes, the majority are in the circa-PHV classification, 71% and 54% for girls and boys, respectively (Table 4). When comparing this proportion with other studies, where only chronological age was used as a reference when selecting talent, a predominance of the post-PHV category was observed [2,15,40]. Therefore, a delay in the chronological age of selection or sports specialization (>15 years) and grouping by bio-banding is proposed in order to avoid biases in sports talent detection processes [6,40,52].

The final selection that enters the CPAP (Table 5) incorporates a significant number of talented athletes that were chosen based on technical criteria (54%), which reinforces the experience and incorporation of sports training professionals during the talent detection processes since they can identify volitional aspects and/or certain fundamental attitudes for a particular sports discipline [2,28,29,30]. According to this evidence, it is valuable to educate coaches and sports professionals in aspects of biological development, relative age, and bio-banding, in order to complement the technical information from a transdisciplinary perspective [2,6,15,28,30,40,52].

The findings and number of children selected in the present study would be in line with the proportion of athletes who finally reach sporting excellence, where the international elite corresponds to 0.0025% of the world population and those classified as world-class athletes to 0.00006%, which would indicate that as time goes by, the number of athletes would decrease [6,53]. According to the technical analysis of our team, in the period 2014–2023, 23% of the beneficiary athletes and/or former beneficiaries of the CPAP were considered in the final list of the Pan American Games Santiago 2023, where the proportion of medals won by this group corresponds to 40.5% of the national total; in addition, 29% of this population has qualified for the Paris 2024 Olympic Games.

This analysis would indicate that the program meets its main objective; however, the work of the present process will begin to be visualized, possibly in 6–8 years. Studies in the area suggest controlling and supporting the process from a multidimensional perspective and avoiding early specialization through the incorporation of other sports activities that allow the acquisition of complementary motor skills, in addition to using competition as an instance of development and learning [2,6].

Finally, from the area of sports projection of the Chilean National Sports Institute, the talent selection process is expected to be consolidated, incorporating a larger school population, a specific selection of sports disciplines by geographical area, and promoting the legacy in infrastructure of the past Pan American Games, Santiago 2023, where one of the fundamental pillars for international sporting success is enhanced [1].

### 4.3. Practical Applications

It is recommended to apply the reference physical and anthropometric assessments, Supplementary Tables S1 and S2, and to consider the coach’s criteria in the support of the selective process from a transdisciplinary perspective and to include grouping models by bio-banding that allows an adequate formation of sports talent [3,6,15,51].

### 4.4. Limitations

The main limitation of the present study is the evaluation of parameters related to cardiovascular endurance, balance, and muscular strength, which are already incorporated by other nations [44,48,49], in addition to extending the selection to Paralympics athletes, who require a differentiated model of detection, according to classification and sport modality. Another potential limitation is that a differentiated analysis by socioeconomic level was not developed, which could affect the number of talents that are effectively detected and achieve high sports performance [5,8]. This last factor will be considered in future reports.

## 5. Conclusions

The present results allow us to know the differences in physical and anthropometric tests in the population of resident schoolchildren in Chile, categorized by sex, geographic zone, and maturity status, which will benefit future talent selection processes in the school context. In addition, the reference percentiles can be applied in the area of physical education by different professionals, not only for high performance but also oriented to motor development in the different biological ages. Through this research, information is provided for the design and implementation of public policies in school physical education, health, and formative sports.

## Figures and Tables

**Table 1:** Total descriptive statistics by sex of the sample studied.

Variable	Total	Girls	Boys
Age (decimal age, years old)	12.08 ±1.52 [3060]	11.96 ±1.50 [1243]	12.16 ± 1.53 [1817]
Weight (kg)	49.3 ± 12.8 [3060]	48.8 ± 11.7 [1243]	49.6 ± 13.5 [1817]
Height (cm)	152.03 ±10.88 [3060]	150.85 ±8.95 [1243]	152.83 ± 11.96 [1817]
BMI (kg/m^2^)	21.08 ±3.88 [3060]	21.26 ±3.83 [1243]	20.96 ± 3.92 [1817]
Maturity status (years at PHV)	−0.84 ± 1.46 [2273]	0.0 ± 1.20 [950]	−1.5 ± 1.32 [1323]
Velocity (m·s^−1^)	5.29 ± 0.60 [2645]	5.11 ± 0.49 [1050]	5.40 ± 0.63 [1595]
SBJ (cm)	148.81± 28.35 [2935]	139.53 ± 24.40 [1199]	155.22 ± 29.11 [1736]
MBCT (cm)	259.10 ± 74.67 [2937]	235.37 ± 55.48 [1192]	275.31 ± 81.46 [1745]
T-test (s)	13.35 ± 1.64 [2576]	13.91 ± 1.51 [1018]	12.98 ± 1.62 [1558]

Data in mean ± standard deviation and *n* for each group in square brackets [ ], BMI (body mass index), PHV (peak height velocity), MBCT (medicine ball chest throw), T-test (agility-T test), RS-30 (thirty-meter running sprint), and SBJ (standing broad jump).

**Table 2:** Planned contrasts by sex and age range in physical and anthropometric tests.

Age Range, *n* (Girls/Boys)	Girls	Boys	Effect Size [C.I]	ANOVA Sex × Age Range (F, *p*-Value [η^2^])
Height (cm)				
A (135/159)	138.70 ± 6.63	137.64 ± 7.36	n.s	F = 35.07, *p* < 0.001 [0.05]
B (230/306)	145.31 ± 6.52	142.31 ± 7.47	0.41 [0.24 to 0.58] †	
C (266/332)	150.05 ± 6.65	148.19 ± 8.23	0.25 [0.09 to 0.42] ұ	
D (241/355)	154.06 ± 6.79	153.83 ± 7.91	n.s	
E (253/426)	156.46 ± 6.56	161.67 ± 8.14	0.71 [0.56 to 0.87] †	
F (118/239)	158.71 ± 6.67	165.64 ± 6.72	0.95 [0.73 to 1.17] †	
RS-30 (m·s^−1^)				
A (105/126)	4.74 ± 0.37	4.88 ± 0.42	0.29 [0.03 to 0.55] ₠	F = 6.77, *p* < 0.001 [0.01]
B (189/256)	4.88 ± 0.38	4.99 ± 0.47	0.21 [0.02 to 0.40] ₠	
C (237/291)	5.06 ± 0.47	5.23 ± 0.50	0.34 [0.16 to 0.51] †	
D (195/319)	5.21 ± 0.43	5.42 ± 0.57	0.42 [0.24 to 0.60] †	
E (224/381)	5.31 ± 0.47	5.68 ± 0.62	0.74 [0.57 to 0.90] †	
F (100/222)	5.46 ± 0.48	5.90 ± 0.57	0.89 [0.65 to 1.12] †	
T-test (s)				
A (100/124)	15.02 ± 1.43	14.59 ± 1.51	0.31 [0.05 to 0.57] ₠	F = 4.80, *p* < 0.001 [0.01]
B (194/255)	14.47 ± 1.48	14.01 ± 1.52	0.33 [0.14 to 0.52] ұ	
C (224/287)	13.96 ± 1.35	13.27 ± 1.42	0.49 [0.31 to 0.66] †	
D (204/316)	13.66 ± 1.39	12.79 ± 1.37	0.62 [0.44 to 0.80] †	
E (207/365)	13.47 ± 1.51	12.27 ± 1.37	0.86 [0.69 to 1.03] †	
F (89/211)	12.95 ± 1.11	11.90 ± 1.23	0.74 [0.50 to 0.99] †	
SBJ (cm)				
A (130/150)	126.17 ± 23.04	134.18 ± 20.43	0.32 [0.08 to 0.55] ұ	F = 8.79, *p* < 0.001 [0.01]
B (224/295)	132.03 ± 22.35	140.07 ± 25.54	0.32 [0.15 to 0.49] †	
C (258/319)	140.04 ± 22.36	148.88 ± 25.74	0.35 [0.19 to 0.52] †	
D (235/343)	142.71 ± 24.64	156.76 ± 27.44	0.56 [0.39 to 0.73] †	
E (242/403)	144.73 ± 23.72	166.98 ± 26.94	0.89 [0.73 to 1.05] †	
F (110/226)	151.20 ± 24.87	174.58 ± 27.38	0.93 [0.71 to 1.16] †	
MBCT (cm)				
A (131/153)	177.56 ± 33.66	197.47 ± 48.73	0.37 [0.14 to 0.60] ұ	F = 34.46, *p* < 0.001 [0.06]
B (227/303)	206.47 ± 46.37	215.01 ± 50.82	n.s	
C (255/327)	224.13 ± 45.36	241.15 ± 60.42	0.32 [0.15 to 0.48] †	
D (230/342)	253.25 ± 47.86	270.95 ± 57.70	0.33 [0.16 to 0.50] †	
E (232/395)	268.16 ± 45.75	333.98 ± 63.85	1.22 [1.06 to 1.38] †	
F (117/225)	280.56 ± 49.21	362.72 ± 66.98	1.52 [1.30 to 1.75] †	

Data in mean ± standard deviation, † (*p* < 0.001), ұ (*p* < 0.01), ₠ (*p* < 0.05), n.s (not significant), C.I [confidence interval, 95%], effect size (Cohen’s d test and C.I), age range [(A = 9 to 9.9, B = 10 to 10.9, C = 11 to 11.9, D = 12 to 12.9, E = 13 to 13.9, and F = 14 to 14.9 years old) and *n* (girls/boys)]. RS-30 (thirty-meter running sprint), T-test (agility T-test), SBJ (standing broad jump), and MBCT (medicine ball chest throw). ANOVA (type III) and interaction for sex and age range (F, *p*-value, and partial eta squared [η^2^]).

**Table 3:** Planned contrasts by geographic area in physical and anthropometric tests by sex.

				Effect Size and Confidence Interval [C.I]			
Dependent Variable	Northern Zone	Central Zone	Southern Zone	North Zone vs. Center Zone	North Zone vs. South Zone	Center Zone vs. South Zone	ANOVA (F, *p*-Value [η^2^])
GIRLS							
Height (cm)	151.55 ± 9.07	150.88 ± 8.48	149.42 ± 9.47	n.s	0.24 [0.09 to 0.39] ұ	n.s	F = 4.68, *p* < 0.01 [0.01]
RS-30 (m·s^−1^)	5.05 ± 0.48	5.22 ± 0.48	5.03 ± 0.49	0.35 [0.21 to 0.48] †	n.s	0.38 [0.22 to 0.54] †	F = 16.06, *p* < 0.001 [0.03]
MBCT (cm)	240.09 ± 57.91	241.32 ± 50.04	213.44 ± 56.51	n.s	0.49 [0.33 to 0.65] †	0.51 [0.36 to 0.67] †	F = 23.69, *p* < 0.001 [0.04]
SBJ (cm)	137.89 ± 23.93	145.43 ± 23.4	130.76 ± 24.23	0.32 [0.19 to 0.44] †	0.30 [0.14 to 0.46] †	0.62 [0.46 to 0.77] †	F = 32.88, *p* < 0.001 [0.05]
T-test (s)	14.02 ± 1.57	13.69 ± 1.46	14.11 ± 1.46	0.22 [0.08 to 0.36] ұ	n.s	0.28 [0.12 to 0.45] ұ	F = 7.27, *p* < 0.001 [0.01]
BOYS							
Height (cm)	154.93 ± 12.05	152.41 ± 11.59	149.87 ± 11.99	0.19 [0.09 to 0.29] †	0.43 [0.29 to 0.57] †	0.24 [0.10 to 0.37] †	F = 20.18, *p* < 0.001 [0.02]
RS-30 (m·s^−1^)	5.38 ± 0.65	5.49 ± 0.62	5.27 ± 0.61	0.19 [0.09 to 0.30] †	0.17 [0.03 to 0.31] ₠	0.37 [0.23 to 0.50] †	F = 15.02, *p* < 0.001 [0.02]
MBCT (cm)	285.96 ± 86.18	278.04 ± 76.28	251.2 ± 79.56	n.s	0.43 [0.29 to 0.57] †	0.36 [0.23 to 0.50] †	F = 19.49, *p* < 0.001 [0.02]
SBJ (cm)	155.85 ± 30.1	159.31 ± 27.67	144.64 ± 27.72	0.14 [0.04 to 0.24] ұ	0.40 [0.26 to 0.54] †	0.54 [0.41 to 0.68] †	F = 31.22, *p* < 0.001 [0.03]
T-test (s)	13.13 ± 1.78	12.65 ± 1.43	13.40 ± 1.55	0.31 [0.20 to 0.42] †	0.17 [0.03 to 0.31] ₠	0.48 [0.34 to 0.62] †	F = 28.21, *p* < 0.001 [0.01]

Data in mean ± standard deviation, † (*p* < 0.001), ұ (*p* < 0.01), ₠ (*p* < 0.05), n.s (not significant). Geographic zone (central zone, northern zone, and southern zone), effect size (Cohen d test and confidence interval [C.I]), north vs. center (north-center zone contrast), north vs. south (north–south zone contrast), center vs. south (center-south zone contrast). RS-30 (thirty-meter running sprint), T-test (agility T-test), SBJ (standing broad jump), MBCT (medicine ball chest throw). ANOVA (type III) one-way (F, *p*-value, and partial eta squared [η^2^]).

**Table 4:** Planned contrasts by maturity status in physical and anthropometric tests in pre-selected athletes of the national talent detection system.

				Effect Size and Confidence Interval [C.I]			
Dependent Variable	Pre-PHV [*n*]	Circa-PHV [*n*]	Post-PHV [*n*]	Pre-PHV vs. Circa-PHV	Circa-PHV vs. Post-PHV	Post-PHV vs. Pre-PHV	ANOVA (F, *p*-Value [η^2^])
GIRLS							
Height (cm)	146.9 ± 7.31 [16]	154 ± 8.11 [102]	156.9 ± 8.26 [26]	0.88 [0.34 to 1.41] ұ	n.s	1.23 [0.61 to 1.86] †	7.73, *p* < 0.001 [0.10]
RS-30 (m·s^−1^)	5.23 ± 0.54 [14]	5.57 ± 0.38 [99]	5.79 ± 0.5 [26]	0.84 [0.26 to 1.42] ұ	0.54 [0.11 to 0.98] ₠	1.38 [0.71 to 2.06] †	8.4, *p* < 0.001 [0.11]
SBJ (cm)	156.2 ± 20.9 [14]	167.9 ± 18.1 [102]	172.5 ± 18.99 [24]	0.63 [0.07 to 1.2] ₠	n.s	0.88 [0.21 to 1.54] ₠	3.5, *p* < 0.05 [0.05]
T-test (s)	12.79 ± 1.47 [16]	12.45 ± 1.03 [101]	11.93 ± 1.2 [25]	n.s	0.49 [0.05 to 0.93] ₠	0.81 [0.17 to 1.44] ₠	3.61, *p* < 0.05 [0.05]
MBCT (cm)	262.5 ± 63.88 [16]	273.5 ± 51.64 [102]	302 ± 49.5 [26]	n.s	0.54 [0.11 to 0.98] ₠	0.75 [0.12 to 1.38] ₠	3.75, *p* < 0.05 [0.05]
BOYS							
Height (cm)	153.49 ± 8.64 [74]	160.6 ± 10.36 [108]	160 ± 12.01 [18]	0.72 [0.42 to 1.01] †	n.s	0.66 [0.14 to 1.18] ₠	11.75, *p* < 0.001 [0.11]
RS-30 (m·s^−1^)	5.85 ± 0.5 [64]	6.08 ± 0.44 [104]	5.92 ± 0.53 [16]	0.48 [0.17 to 0.80] ұ	n.s	n.s	4.82, *p* < 0.01 [0.05]
SBJ (cm)	183 ± 22.02 [73]	190.6 ± 20.17 [106]	190.2 ± 22.2 [18]	n.s	n.s	n.s	2.95, *p* > 0.05 [0.03]
T-test (s)	11.79 ± 1.04 [72]	11.34 ± 1.03 [108]	11.40 ± 0.99 [18]	0.44 [0.14 to 0.74] ₠	n.s	n.s	4.23, *p* < 0.05 [0.04]
MBCT (cm)	306.8 ± 62.85 [74]	344.4 ± 77.49 [107]	356.3 ± 92.15 [18]	0.51 [0.21 to 0.81] ұ	n.s	0.67 [0.15 to 1.19] ₠	6.79, *p* < 0.01 [0.06]

Data in mean ± standard deviation, † (*p* < 0.001), ұ (*p* < 0.01), ₠ (*p* < 0.05), n.s (not significant), effect size (Cohen’s d test and confidence interval [C.I]). PHV (peak height velocity), pre-PHV (−0.5 years at PHV), circa-PHV (≥−0.5 years and ≤0.5 years at PHV), and post-PHV (>0.5 years at PHV). RS-30 (thirty-meter running sprint), T-test (agility T-test), SBJ (standing broad jump), and MBCT (medicine ball chest throw). ANOVA (type III) one-way (F, *p*-value, and partial eta squared [η^2^]).

**Table 5:** Physical and anthropometric variables, according to selection criteria of the national talent detection system.

Variable (n1, n2)	Selected by Physical Test	Selected by Technical Criteria
GIRLS		
Decimal age (years old) (12, 15)	12.34 [11.46–13.31]	12.05 [11.05–13.36] n.s
Maturity status (years at PHV) (12, 13)	0.0 [0.0–0.34]	0.0 [0.0–0.0] n.s
Height (cm) (12, 15)	154.5 [151.76–159.38]	152 [143–157.5] n.s
SBJ (cm) (12, 15)	171.5 [160.25–190.50]	149 [125.5–155] ұ
RS-30 (m·s^−1^) (12, 15)	5.75 [5.45–5.97]	5.30 [5.15–5.43] ₠
T-test (s) (12, 14)	12.52 [11.89–12.80]	14.44 [13.02–15.14] ₠
MBCT (cm) (12, 15)	306 [259.75–329.75]	222 [196.50–259] ұ
BOYS		
Decimal age (years old) (19, 21)	12.71 [11.69–13.84]	12.8 [10.99–13.70] n.s
Maturity status (years at PHV) (17, 18)	0.0 [−0.32–0.0]	0.0 [−0.38–0.0] n.s
Height (cm) (19, 21)	160 [148.50–164.75]	160 [148–166] n.s
SBJ (cm) (18, 20)	191 [165–209.5]	165.5 [135–187.5] ₠
RS-30 (m·s^−1^) (19, 21)	6.07 [5.56–6.41]	5.64 [4.88–6.57] n.s
T-test (s) (19, 21)	11.81 [10.75–12.93]	12.94 [11.78–14.06] ₠
MBCT (cm) (19, 21)	330 [254–375.5]	320 [239–380] n.s

Mann–Whitney–Wilcoxon statistical test. ұ (*p* < 0.01), ₠ (*p* < 0.05), n.s (not significant). Data in median and interquartile range [IQR]. n1 and n2 (n physical/anthropometric test group and technical criterion, respectively). PHV (peak height velocity), RS-30 (thirty-meter running sprint), T-test (agility T-test), SBJ (standing broad jump) and MBCT (medicine ball chest throw).

## Data Availability

The raw data supporting the conclusions of this article will be made available by the authors upon request.

## Supplementary Materials

The following supporting information can be downloaded at: https://www.mdpi.com/article/10.3390/jfmk10010006/s1, Table S1. Percentile values by maturity status. Table S2. Percentile values by age range.
