# Supplementary material for: Chilean National Sports Talent Detection System: Influence of Biological Age, Sex, and Geographic Area
Source: J Funct Morphol Kinesiol. 2024 Dec 27;10(1):6. doi: 10.3390/jfmk10010006 (PMC11755584; doi:10.3390/jfmk10010006)
Supplement: Supplementary file 1 [file jfmk-10-00006-s001.zip › jfmk-3200024-supplementary.pdf]

Journal of
Functional Morphology and Kinesiology

MDPI

Table S1. Percentile values by maturity status.

GIRLS									BOYS							
**N**	**10th**	**15th**	**25th**	**50th**	**75th**	**85th**	**90th**	**Maturity status**	**N**	**10th**	**15th**	**25th**	**50th**	**75th**	**85th**	**90th**
Height (cm)																
159	137	138.6	141	146	151	153.4	155	pre-PHV	516	138	140	143	150	157	160	162
700	138.5	142	145	151.3	157	160	161.2	circa-PHV	880	138	140	145	156	165	168	169.7
200	148	150	152	155	159	163	166	post-PHV	117	140.8	143	150	162	169	171	174
RS-30 ($m·s^{-1}$)																
132	4.19	4.28	4.55	4.87	5.16	5.30	5.39	pre-PHV	456	4.51	4.65	4.88	5.26	5.64	5.88	6.04
618	4.51	4.62	4.76	5.06	5.38	5.55	5.66	circa-PHV	837	4.62	4.77	4.96	5.39	5.84	6.12	6.27
184	4.74	4.84	4.96	5.29	5.6	5.77	5.88	post-PHV	106	4.90	4.97	5.21	5.52	5.98	6.19	6.26
SBJ (cm)																
155	97.4	102.1	111	132	148	160	163	pre-PHV	499	114	120	131	150	170	180	187
681	110	115	123	140	156	164	171	circa-PHV	848	120	126	135	155	177	190	197
193	120	122	130	143	159	170	181.6	post-PHV	115	127	136	149.5	167	184.5	193	199
MBCT (cm)																
150	150	154.4	170	200	237.8	272	280	pre-PHV	492	170	187.7	208	250	299.3	320	340
680	160	170	190	230	270	290	300	circa-PHV	853	173.2	190	220	277	342	373	391
188	212	220	230	263.5	300	311	327.2	post-PHV	112	203	222	249	320	371.3	404.8	420
T-test (s)																
131	12.69	12.82	13.22	14.39	15.50	16.36	16.81	pre-PHV	470	11.26	11.53	12.00	13.04	14.16	14.74	15.29
620	12.13	12.38	12.85	13.88	14.98	15.50	16.01	circa-PHV	801	10.96	11.26	11.71	12.75	13.91	14.51	15.05
174	11.63	11.95	12.45	13.21	14.24	14.76	15.17	post-PHV	105	10.86	11.17	11.51	12.29	13.38	13.95	14.64

Pre-Phv (< -0.5 years from PHV), circa-PHV (≥ -0.5 years and ≤ 0.5 years to PHV) and Post-Phv (>0.5 years to PHV). PHV (Peak height velocity). RS-30 (thirty-meter running sprint), T-test (agility-T test), SBJ (Standing broad jump test) and MBCT (Medicine ball chest throw).

Table S2. Percentile values by age range

GIRLS							age	BOYS							
**N**	**10th**	**15th**	**25th**	**50th**	**75th**	**85th**	**90th**	**range**	**N**	**10th**	**15th**	**25th**	**50th**	**75th**	**85th**	**90th**
Height (cm)																
135	130.4	132.4	135.0	138.0	143.0	145.0	147.0	A	159	129.3	130.0	133.0	137.0	142.0	145.0	147.0
230	137.0	138.0	141.1	145.1	149.8	152.0	154.1	B	306	133.0	134.8	138.0	142.0	147.0	150.1	152.8
266	141.2	143.0	145.0	150.1	154.3	156.9	158.0	C	332	138.0	140.0	142.0	147.9	154.0	157.0	159.0
241	145.8	148.0	150.0	154.0	158.0	160.0	162.0	D	355	144.0	146.0	149.0	153.9	159.0	162.0	164.0
253	149.0	150.0	152.7	156.0	160.0	163.5	165.0	E	426	151.0	153.8	157.0	162.0	168.0	170.0	172.0
118	150.3	151.3	154.1	159.0	164.0	166.0	166.9	F	239	157.0	159.5	161.6	166.0	169.1	172.0	174.0
RS-30 ($m \cdot s^{-1}$)																
105	4.19	4.26	4.50	4.74	5.04	5.15	5.23	A	126	4.25	4.44	4.62	4.92	5.18	5.30	5.42
189	4.43	4.52	4.65	4.88	5.17	5.28	5.36	B	256	4.38	4.50	4.62	5.02	5.35	5.47	5.57
237	4.44	4.57	4.76	5.02	5.38	5.56	5.66	C	291	4.55	4.73	4.88	5.25	5.54	5.70	5.84
195	4.64	4.77	4.89	5.16	5.51	5.63	5.82	D	319	4.68	4.84	5.03	5.42	5.77	5.94	6.16
224	4.66	4.74	4.95	5.31	5.66	5.80	5.92	E	381	4.91	5.02	5.25	5.68	6.12	6.32	6.48
100	4.89	5.00	5.14	5.39	5.70	5.89	6.07	F	222	5.14	5.31	5.60	5.94	6.30	6.43	6.56
SBJ (cms)																
130	93.5	101.4	112.3	127.5	140.0	145.0	153.3	A	150	110.0	115.0	120.0	133.0	146.8	155.7	161.1
224	100.3	109.0	114.8	133.0	145.3	153.0	158.7	B	295	109.0	116.0	123.0	138.0	156.0	164.0	173.6
258	112.7	119.6	125.0	140.0	154.8	162.0	169.3	C	319	114.0	120.0	131.5	150.0	165.0	175.3	180.2
235	111.4	117.1	124.0	144.0	159.0	166.9	173.2	D	343	124.2	130.0	135.5	158.0	175.0	185.0	192.8
242	114.0	120.0	129.0	142.5	162.0	169.9	176.0	E	403	132.2	138.3	148.0	167.0	187.0	195.7	200.8
110	120.6	130.0	135.3	151.0	166.0	174.0	186.0	F	226	140.0	145.8	154.3	174.0	195.0	205.3	212.0
MBCT (cms)																
131	140.0	150.0	150.0	170.0	200.0	210.0	220.0	A	153	136.0	150.0	160.0	198.0	225.0	246.0	260.0
227	150.0	160.0	174.0	200.0	237.5	255.0	272.0	B	303	150.0	157.0	174.5	215.0	250.0	270.0	280.0
255	168.0	175.5	190.0	220.0	254.5	270.0	289.6	C	327	166.2	180.0	200.0	240.0	280.0	300.2	318.8
230	190.0	200.0	218.0	254.5	282.8	300.0	310.0	D	342	195.0	210.0	231.3	265.0	303.5	333.0	350.0
232	220.0	220.0	232.8	260.0	297.3	310.0	326.9	E	395	252.0	267.0	288.0	328.0	374.5	400.0	420.0
117	222.4	231.2	240.0	278.0	305.0	330.0	350.4	F	225	276.4	295.2	320.0	365.0	402.0	430.4	448.0

Table S2. continued

T-test (s)															
100	13.31	13.53	14.15	14.87	16.07	16.81	17.00	A	124	12.85	13.06	13.54	14.50	15.60	16.44	16.82
194	12.83	12.97	13.42	14.29	15.41	16.20	16.80	B	255	12.08	12.40	12.84	13.94	15.00	15.70	16.51
224	12.24	12.46	12.95	13.95	14.99	15.29	15.88	C	287	11.54	11.84	12.21	13.18	14.13	14.79	15.31
204	12.05	12.30	12.66	13.45	14.73	15.26	15.56	D	316	11.11	11.40	11.82	12.57	13.72	14.32	14.78
207	11.49	11.83	12.34	13.37	14.44	15.16	15.42	E	365	10.72	10.94	11.30	12.03	13.16	13.76	14.17
89	11.82	11.95	12.20	12.95	13.69	14.17	14.33	F	211	10.39	10.62	11.04	11.78	12.66	13.17	13.63

Age range [A= 9 to 9.9 years, B= 10 to 10.9 years, C=11 to 11.9 years, D=12 to 12.9 years, E= 13 to 13.9 years, F= 14 to 14.9 years]. RS-30 (thirty-meter running sprint), T-test (agility-T test), SBJ (standing broad jump test) and MBCT (Medicine ball chest throw).
